# The influence of streamers’ physical attractiveness on consumer response behavior: based on eye-tracking experiments

**DOI:** 10.3389/fpsyg.2023.1297369

**Published:** 2024-01-12

**Authors:** Xiaoli Tang, Zefeng Hao, Xiaolin Li

**Affiliations:** School of Economics and Management, Yanshan University, Qinhuangdao, China

**Keywords:** streamers, physical attractiveness, quasi-social interaction, flow experience, consumer response, consumer involvement

## Abstract

Live streaming marketing has become a popular topic of academic research. However, relatively few studies have been conducted in terms of the physical attractiveness of streamers, and even fewer studies have analyzed the changes in cognitive-emotional mechanisms that affect consumer response behavior. Based on SOR theory and cognitive-emotional system theory, this study proposes a theoretical research model and analyzes the internal mechanism of streamers’ physical attractiveness affecting consumers’ response using a combination of eye-tracking experiments and questionnaires. The results showed that: compared to streamers with lower physical attractiveness, consumers pay longer attention to streamers and products with higher physical attractiveness, and their response behaviors (continued watching intention, continued engagement intention, and purchase intention) are more active; compared to consumers with low involvement, consumers with high involvement pay longer attention to the product and perceive the process for a longer period of time; and quasi-social interaction and the flow experience play the role of a chain mediator between streamers’ physical attractiveness and consumers’ response behaviors. This study not only has certain theoretical significance for expanding the applicable boundaries of the physical appearance halo effect, but also has important practical significance for live broadcasting e-commerce companies to effectively adopt visual marketing and enhance customer retention.

## 1 Introduction

With the increase in Internet penetration and the increasing improvement of online sales platforms, live-streaming marketing has emerged. According to the “50th China Internet Development Statistical Report,” the penetration rate of live e-commerce users in 2022 was as high as 65.5%, with a user scale of 469 million, and the trend is continuing to grow. The rapid development of live-streaming marketing has made streamers a competitive profession, and the low entry threshold has led to uneven quality of streamers in the existing live marketing market. For consumers, streamers have the role of recommending guidance, interactive display, but also one of the important factors that prompts them to make consumption decisions (Lin et al., 2021; Li et al., 2022). As a result, the selection and cultivation of streamers has become one of the issues that live e-commerce companies should consider.

In the process of interpersonal communication, appearance is one of the most easily captured information, similarly, in the process of e-commerce live broadcasting, the easiest way to attract consumers’ eyes is the appearance of the streamer. This feature affects the differences in others’ judgments of an individual’s ability, character, and degree of preference, and creates differences in expectations and perceptions. This phenomenon is known as appearance stereotyping (Agthe et al., 2014). Numerous studies have shown that beautiful people can receive more attention and are motivated to give more positive affective feedback from others, which in turn triggers approach behavior (Burns and Farina, 1987). As a result, consumers show more positive response behaviors toward the beautiful. This potential advantage due to beauty has been described as the “beauty is good” stereotype effect. Currently, research on this effect has focused on the areas of investment management, human resources, and consumer behavior. In the area of consumer behavior, most studies have been conducted on service personnel and endorsed models, while there is still a gap in research on the influence of streamers’ physical attractiveness on consumer behavior. Therefore, the first question this study intends to explore is: does the physical attractiveness of streamers have an impact on consumer behavior?

Most of the previous studies on live streaming marketing have focused on changes in consumers’ single cognitive or affective systems, e.g., social presence (Sun et al., 2021; Chen and Liao, 2022; Liu et al., 2022), psychological contract (Hsu, 2023), quasi-social interaction (Xu et al., 2020), flow experience (Cui et al., 2022), pleasure and arousal (Li et al., 2022; Tong et al., 2022). Existing research still lacks empirical analyses that take consumer cognitive-emotional systems as the focus of research and delve into the relationship between streamers and consumer response behaviors. Cognitive-emotional system theory suggests that individuals, under the stimulating effect of the external environment, will produce rational cognitive and emotional impulse responses, and ultimately affect individual behavior. Cognitive and affective systems interact with each other, and the psycho-cognitive processes produced by individuals after receiving external stimuli are often accompanied by emotional arousal (Mischel and Shoda, 1995). Since quasi-social interaction and flow experience belong to cognitive and affective factors, respectively, and it has been shown that quasi-social interaction has an impact on consumers’ arousal emotion (Xu et al., 2020). Therefore, this study hypothesizes that there is an intrinsic connection between quasi-social interaction and flow experience. The second question to be explored in this paper is: what is the mechanism of action between the physical attractiveness of streamers and consumer response behavior?

ELM (The Elaboration Likelihood Model) states that individuals transform information through central and marginal processing paths. Consumer involvement affects individuals’ choice of information processing paths, and existing studies have shown that consumer involvement plays a moderating role in influencing consumer behavior (Park and Lee, 2008). Consumers with low involvement are more likely to process information through marginal paths and rely more on cognitive tendencies generated by external stimuli to make consumption decisions. Therefore, the third question to be explored in this study is: how does consumer involvement modulate the role of streamers’ physical attractiveness on consumer response behavior?

Relevant studies in psychology agree that eye movements can reflect the allocation of subjects’ visual attention (Rayner, 1998), and eye-tracking technology can more accurately describe eye movement data (Scheiter and Van Gog, 2009), which has been widely used in the fields of psychology, news communication and consumer behavior. Previous studies related to streamers were more often conducted in the form of questionnaires, which were often used to collect and measure data by way of respondents’ hindsight recall, resulting in researchers being unable to assess the real-time situation of consumers when watching live broadcasts. Therefore, focusing on the attractiveness of streamers’ physical appearance to consumers from the perspective of visual attention can further understand the potential psychological cognitive process of consumers. In addition, the study showed that consumer eye movements can help marketers capture information such as consumer attention and their product choice (Pieters and Warlop, 1999). Thus, pre-exploring the fourth question of this study: What are the links between streamers’ physical attractiveness, consumers’ attention duration and consumer response behavior, and what role does consumer involvement play? In conclusion, this study intends to use a combination of eye tracking and behavioral data to deeply explore the influence of streamers’ physical attractiveness on consumers’ response behavior, exploring the effects of different physical attractiveness of streamers on consumers’ visual attention process (perceptual process) by means of eye-tracking, and exploring the effects of different physical attractiveness of streamers on consumers’ psychological cognitive outcomes and behavioral decision-making outcomes (perceptual outcomes) by means of questionnaire data.

In summary, based on S-O-R theory and cognitive-emotional system theory, this study intends to explore the influence of streamers’ physical attractiveness on consumers’ response behaviors in the process of live streaming marketing through eye-tracking experiments. This study intends to construct a model of the mechanism of action based on quasi-social interaction and flow experience. It also introduces consumer involvement as a moderating variable to explore the role of consumer traits in the purchase decision process. The results of this research will help to expand the research scope of physical attraction, and can provide suggested theoretical support and practical suggestions for improving the customer retention index of live broadcasting and promoting the sales growth of live broadcasting products.

## 2 Literature review and research hypotheses

In the live-streaming marketing context, the streamer is the main body that brings cognitive and emotional value to consumers. Current research related to streamers has found that streamer’s traits (professionalism, real-time interactivity, attractiveness, and image matching) directly affect consumer’s perceived value, which in turn has an impact on their purchase intention (Zhou and Huang, 2023). Guo et al. (2022) classified streamers’ attractiveness, competence, and communication style as beauty, warmth, expertise, humor, and passion, exploring the impact of these characteristics on consumers’ behavioral intentions through the mediating role of their perceived value (utility and hedonic). In addition, other scholars focus on a particular characteristic of streamers, and the study confirmed that the interactivity of network star streamers have an impact on consumers’ purchase intention through social presence and flow experience (Sun et al., 2021). All of the above studies have explored the influence of one or more attributes of streamers on consumers’ purchasing decisions. However, these studies have not deeply explored the most intuitive trait of streamer’s physical attractiveness, and have not broken down the cognitive path of consumer information processing. Similarly, these studies have not deeply analyzed the micro-mechanisms of how consumers’ cognitive-emotional system influences their response behaviors when they are stimulated by the streamers’ appearance.

**Figure 1:**
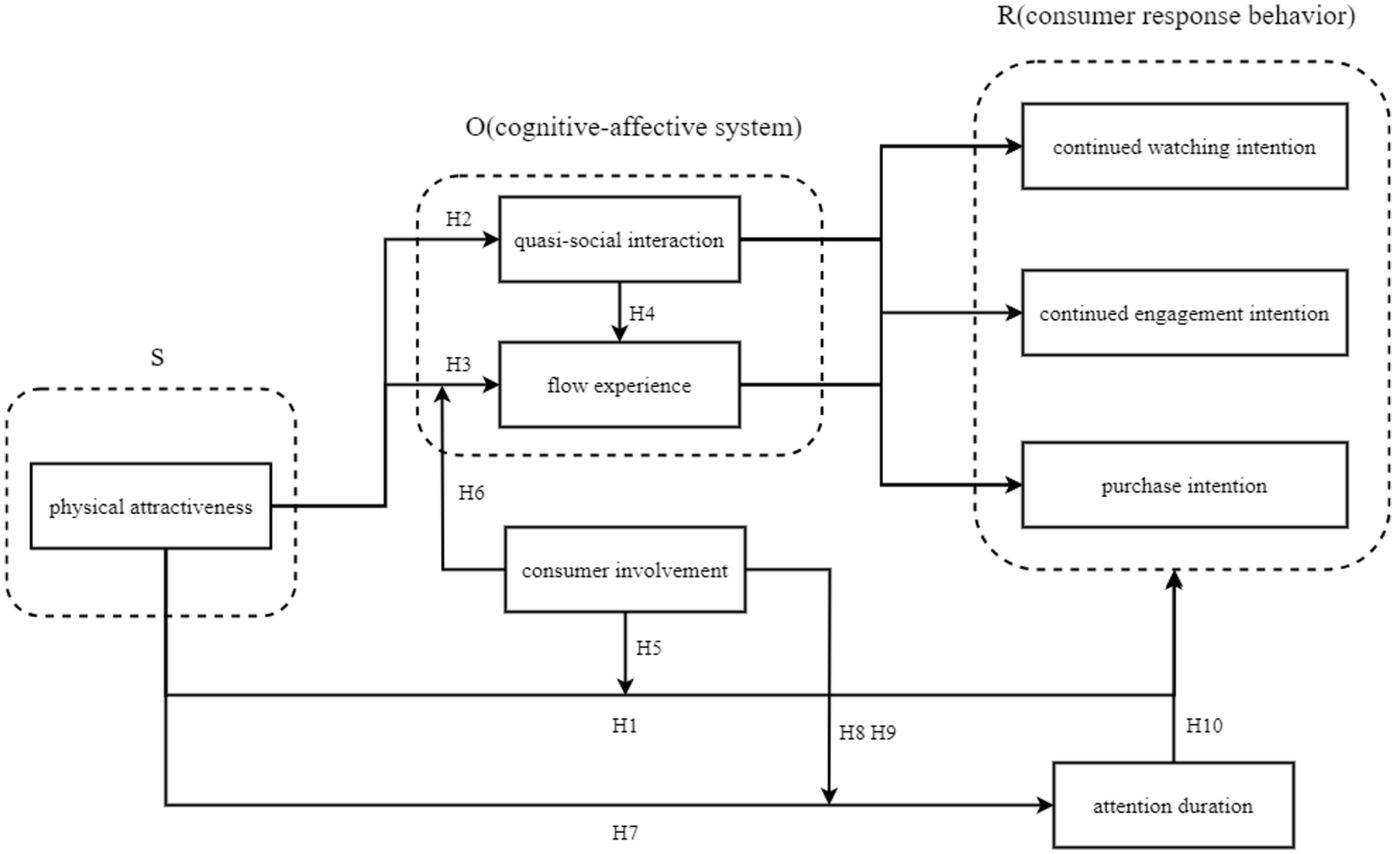
The theoretical framework.

Compared with traditional e-commerce, live e-commerce can provide more vivid explanation services and visual experience, so that consumers and streamers can produce emotional connection. Under the influence of “all people love beauty,” live broadcast e-commerce can attract consumers’ eyes by choosing streamers who have the advantage of appearance to explain the products. Therefore, consumers have a greater likelihood of continuing to watch the live broadcast, so that consumer attention can be converted into e-commerce marketing revenue. For individual consumers, whether the more aesthetic visual experience can attract consumers’ attention, whether it can trigger their positive cognitive and emotional responses, especially whether it can enhance their continued watching, engagement and subsequent purchase intention, are all topics worthy of in-depth exploration. Figure 1 shows the theoretical framework.

### 2.1 S-O-R theory

S-O-R (Stimulus-Organism-Response) theory was initially proposed and applied to environmental psychology research by Russell, and Jacoby subsequently expanded it to the field of consumer behavior. Donovan and Rossiter first applied the model to retail settings. To date, the S-0-R model has been widely used in a variety of retail studies in online and offline environments (Donovan and Rossiter, 1982) to understand consumer behavior, including e-commerce live streaming (Liu et al., 2022), online reviewing (Bigne et al., 2020), and mobile travel (Fang et al., 2017). Among them, stimulus means “external influences that evoke an individual.” “Organism” refers to the individual’s emotional and cognitive responses to the surrounding environment, the cognitive state represents the consumer’s mental processes, including everything in the consumer’s mind related to the acquisition, processing, retention, and retrieval of information; and the affective state reflects the consumer’s emotions such as excitement and pleasure in response to environmental stimulus. “Response” refers to the approach and avoidance behaviors of stimulus and organism. In summary, the theory describes the positive (approach behavior, including positive communication, willingness to buy, willingness to browse, etc.) or negative (avoidance behavior, including negative communication, lack of willingness to buy/stay, etc.) responses of an individual through environmental cues, affective, and cognitive processes (Bitner, 1992; Eroglu et al., 2001; Floh and Madlberger, 2013).

### 2.2 Cognitive-affective theory

Cognitive-Affective Theory was proposed by Mischel in the 1970s. The theory states that stimulus from external situations interact with the complex cognitive-affective units (CAUs) in an individual’s system, prompting rational cognitive or affective impulsive responses and ultimately influencing the individual’s behavioral choices (Mischel, 1973). The theory consists of two major perspectives: first, a single cognitive or affective system within an individual is activated in a given situation, which in turn leads to individual behavior; second, an individual’s internal cognitive and affective systems are activated in a given situation, which manifests situational factors activate the individual’s cognitive system, which furthermore evokes the affective system and ultimately influences the emergence of the individual’s attitudes and behaviors (Mischel and Shoda, 1995). Based on the second viewpoint, this study explores the effect of streamers’ physical attractiveness on consumers’ cognitive and affective systems. This theory has been applied by some scholars in the field of consumer behavior. For example, Dai et al. (2020) investigated how perceived information overload (cognition) affects social media users’ information avoidance intentions through fatigue, frustration, and dissatisfaction (affect). Loh et al. (2022) investigated the cognitive (mobile usefulness, price retention, and relevant network size)-affective (satisfaction and technological stress) linkages of consumers’ payment continuance intention during the COVID-19 period.

### 2.3 Halo effect

Halo effect refers to an individual’s tendency to form an overall impression of another person based on positive or negative partial impressions, this preference phenomenon discovered by Thorndike in 1920 (Lachman and Bass, 1985). A prime example of the halo effect is the influence of physical attractiveness on an individual’s perception, which is known as the “beautiful is good” effect (Dion et al., 1972). Specifically, attractive individuals are often perceived as having more desirable traits, for example, such individuals are perceived as more confident, emotionally stable, intelligent, responsible, sociable, and trustworthy (Batres and Shiramizu, 2023), and as a result, they are treated more positively than unattractive individuals (Efran, 1974; Landy and Sigall, 1974; Langlois et al., 2000).

### 2.4 The effect of streamers’ physical attractiveness on consumer response behavior

SOR theory suggests that external stimuli can influence an individual’s internal state, which in turn triggers his or her behavioral response (Cui et al., 2022). In the live-streaming shopping context, the streamer acts as an information transmitter, which directly affects consumers’ information reception and behavioral decisions. Attractiveness is considered to be the first step in establishing a relationship (Zheng et al., 2020). Extrinsic attractiveness refers to the degree to which the target person is pleasant and is crucial in the online socialization process (Lo, 2008). During interpersonal interactions, due to the halo effect of physical appearance, lookers are more likely to obtain higher willingness to interact from others and tend to inspire more positive emotional responses from others (Korabik, 1981). It has been shown that the appearance attractiveness of service workers can have a positive impact on customer response, Argo et al. (2008) found that service workers with higher appearance attractiveness resulted in higher customer satisfaction and customer purchase intention; Customers tend to choose attractive salespeople and are more likely to respond to their sales pitches, ultimately demonstrating a higher purchase intention (DeShields et al., 1996). Therefore, streamers who have a pleasing appearance and whose external stimuli agree with consumers’ aesthetics will lead to more positive response behaviors, i.e., continued watching intention (Chen and Liao, 2022), continued engagement intention (Lv et al., 2022), and purchase intention (Liu et al., 2022; Dong X. et al., 2023).

In summary, the physical attractiveness of streamers can be used as an external stimulus, which in turn affects consumers’ behavioral responses. Therefore, the following hypotheses are proposed in this study:

*H1*: Streamers with high physical attractiveness elicit more positive response behavior from consumers than those with low physical attractiveness.

*H1a*: Consumers have a more positive continued watching intention in the face of a streamer with high physical attractiveness compared to a streamer with low physical attractiveness.

*H1b*: Consumers have a more positive continued engagement intention in the face of a streamer with high physical attractiveness compared to a streamer with low physical attractiveness.

*H1c*: Consumers have a more positive purchase intention in the face of a streamer with high physical attractiveness compared to a streamer with low physical attractiveness.

### 2.5 The mediating role of quasi-social interaction and flow experience

Quasi-social interaction has been defined as the extent to which media users perceive media characters as close social partners (Horton and Richard Wohl, 1956), and it is an illusion created by viewers to have face-to-face conversations with media characters through the medium and to want to get as close as possible to the media characters in reality (Kim and Song, 2016). In the context of live-streaming marketing, quasi-social interaction is defined as the intention of consumers who are attracted to the streamer during their participation in the live shopping process and want to get to know and interact with the streamer in reality. Predictors of quasi-social interaction include media characters’ attractiveness in terms of socialization and appearance (Perse and Rubin, 1989). Rubin and Step (2000) found that media characters’ interpersonal similarity and attractiveness affect viewers’ perceived quasi-social interaction, and the two are positively correlated. In addition, some studies confirmed that quasi-social interaction play a mediating role between media personalities and consumers’ purchase intentions. Lee and Watkins (2016) showed that YouTube platform users’ perceived quasi-social interaction with video bloggers positively affect their luxury goods purchase intentions; the effect of viewers’ self-congruence and value congruence with the hosts of hotel livestreaming program on consumers’ purchase intentions were mediated by quasi-social interaction (Shen et al., 2022). Therefore, in the live-streaming marketing context, the appearance of streamers can attract consumers’ attention and affect their quasi-social interaction perception, which in turn affects their response behavior.

Csikszentmihalyi proposed the concept of flow experience in 1975, which refers to a psychological state in which an individual forgets the existence of time, ignores the surrounding environment, loses self-consciousness, and enjoys himself or herself because of his or her total dedication to something (Csikszentmihalyi, 1988). This concept is widely used in sports, education, website design, and consumer behavior. In the context of live-streaming marketing, the flow experience is defined as a psychological state in which consumers are fully engaged in the live streaming situation when watching live e-commerce broadcasts, and are attracted to and immersed in the live streaming environment (the streamer, the product, and the live streaming background, etc.). In previous studies, the antecedent influences of the flow experience can be categorized into two aspects: external environmental characteristics and consumers’ characteristics (Dong et al., 2010; Cui et al., 2022; Tian and Ou, 2023). Website attractiveness, speed and interactivity as external environment features are directly related to the viewer’s flow experience (Skadberg and Kimmel, 2004). And streamer physical attractiveness, as an external trait of the streamer and as an external environmental stimulus in the live-streaming context, is one of the influencing factors acting on the consumers’ flow experience. In addition, existing research showed that the flow experience has a direct positive effect on both consumer behavioral intention and consumption behavior. When consumers browse on the relevant website, the resulting flow experience can further influence consumers’ intention to purchase products from the website and their intention to visit and use the website again (Luna et al., 2003). Dong et al. (2010), Dong W. W. et al. (2023), and Dong X. et al. (2023) found that website characteristics (interaction, entertainment, and service quality), streamer attributes (credibility, professionalism, attractiveness, and interactivity), and consumer purchase intention were mediated by the flow experience. Therefore, in a live marketing situation, streamers attract consumers through their appearance advantage, prompting them to immerse themselves in the live broadcast, generate a mind-flow experience, and then stimulate consumers to generate responsive behaviors.

Based on the SOR theory and the above compendium, this study takes the physical attractiveness of streamers as an external stimulus (S), quasi-social interaction, and flow experience as changes in consumers’ own internal states (O), and consumer responses as their subsequent behavioral responses (R). The following hypotheses are ultimately proposed:

*H2*: Quasi-social interaction mediates the relationship between streamers' physical attractiveness and consumer response.

*H2a*: Quasi-social interaction mediates the relationship between streamers' physical attractiveness and consumers' continued watching intention.

*H2b*: Quasi-social interaction mediates the relationship between streamers' physical attractiveness and consumers' continued engagement intention.

*H2c*: Quasi-social interaction mediates the relationship between streamers' physical attractiveness and consumers' purchase intentions.

*H3*: Flow experience mediates the relationship between streamers' physical attractiveness and consumers' response.

*H3a*: Flow experience mediates the relationship between streamers' physical attractiveness and consumers' continued watching intention.

*H3b*: Flow experience mediates the relationship between streamers' physical attractiveness and consumers' continued engagement intention.

*H3c*: Flow experience mediates the relationship between streamers' physical attractiveness and consumers' purchase intention.

### 2.6 Chain mediation of quasi-social interaction and flow experience

Cognitive-affective systems theory suggests that stimuli from the external environment act on an individual’s cognitive and affective systems and ultimately affect their own behavior, either by a single cognitive or affective unit acting or by both units interacting with each other (Mischel and Shoda, 1995). It has been found that the psycho-cognitive processes produced by individuals after receiving external stimuli are often accompanied by emotional arousal (Xu et al., 2020). In a live marketing situation, certain factors in the live broadcasting process induce consumers’ cognitive and subsequent emotional responses. For example, the consumer’s self-congruence and value congruence with the host of hotel livestreaming program increased the consumers’ perception of the host’s quasi-social interaction (cognitive unit), which triggers his or her emotional engagement (affective unit), and finally acts on the consumer’s purchase intention (Shen et al., 2022); live streaming contextual matching positively affects the consumer’s cognitive process (perceived trust and perceived value), and then subsequently has an impact on the consumer’s affective process (perceived pleasure), which in turn facilitates the consumer’s purchase intention generation (Shang et al., 2023). Dong W. W. et al. (2023) and Dong X. et al. (2023) showed that social presence (cognition) and flow experience (emotion) stimulate consumers’ purchase intention in live-streaming marketing contexts.

To summarize, quasi-social interaction is the interpersonal association between consumers and media characters perceived by consumers, which belongs to the cognitive unit in the individual cognitive-emotional system; whereas the flow experience is an emotional state manifested by consumers, which belongs to the emotional unit in the individual emotional cognitive system. Therefore, in the context of live marketing, the physical attraction of streamers will prompt consumers to want to establish a social relationship with the streamer, which in turn triggers a change in the emotional state of the consumer, resulting in a flow experience in which the consumer is immersed and oblivious, and ultimately the consumer will follow the streamer’s guidance to produce more positive response behavior. Based on the above analysis, this study proposes the following hypotheses:

*H4*: Quasi-social interaction and flow experience act as chain mediators between streamers' physical attractiveness and consumers' response.

*H4a*: Quasi-social interaction and flow experience act as chain mediators between streamers' physical attractiveness and consumers' continued watching intention.

*H4b*: Quasi-social interaction and flow experience act as chain mediators between streamers' physical attractiveness and consumers' continued engagement intention.

*H4c*: Quasi-social interaction and flow experience act as chain mediators between streamers' physical attractiveness and consumers' purchase intention.

### 2.7 The moderating effect of consumer involvement

Consumer involvement is defined as the degree to which a consumer pays attention to something based on his or her intrinsic interests, values, and needs (Zaichkowsky, 1985), which is usually related to the consumer’s personality and emotions, or to the perceived importance of the subject of attention and the stimulus (Park and Lee, 2008). Research has shown that the generation of consumer behavioral decisions is closely related to the level of involvement. The ELM states that individuals have two paths for processing information, the center path and the edge path. When consumers have the ability and motivation to scrutinize and think deeply about the details of thematic information, they will actively search for information and devote more cognitive resources to synthesize and process the information in a detailed way, which relies on the central path; on the contrary, consumers will be influenced by simple cues in the context, such as associations generated by the thematic information or a certain kind of emotional stimulation, which follows the edge path (Cacioppo and Petty, 1982). In the live marketing context, a beautiful streamer can trigger positive emotions in consumers. Consumers with a higher degree of involvement tend to think rationally about the product information, so the cognitive tendency triggered by external stimuli will be weakened by their rational purchasing decisions; while consumers with a lower degree of involvement rely more on the emotional changes caused by the appearance of the streamer to make consumption decisions. In addition, the consumers’ flow experience relies on consumers’ personal traits. For example, Koufaris (2002) confirmed that consumers’ product involvement level has the greatest influence on the flow experience; Csikszentmihalyi (1988) and Teng (2011) found that individual personality traits and temperament affect their own flow experience, confirming that people with curiosity, endurance, and self-transcendence traits are more likely to have a flow experience。.

Based on this, this study argues that both the streamer’s physical attractiveness and consumer involvement will have an impact on consumers’ flow experience and response behavior in a live marketing context. And the following hypotheses are proposed:

*H5*: Consumer involvement significantly influences the relationship between streamers' physical attractiveness and consumers' response behavior.

*H5a/H5b/H5c*: When consumer involvement is low, Streamers with high physical attractiveness can trigger consumers more positive continued watching/continued engagement/purchase intention.

*H5d/H5e/H5f*: When consumer involvement is high, the effect of streamers with high physical attractiveness on consumers’ continued watching/continued engagement/purchase intention is not significant.

*H6*: Consumer involvement significantly influences the relationship between streamers' physical attractiveness and flow experience.

*H6a/H6b/H6c*: Consumer involvement effectively moderates the relationship between streamers' physical attractiveness and consumers' continued watching/continued engagement/purchase intention through the mediating role of flow experience.

### 2.8 Eye-tracking technology

Eye-tracking technology, a non-invasive, simple and innovative cognitive neuroscience technique, is commonly used to explore consumers’ visual attention allocation and cognitive processes in response to a given stimulus (Just and Carpenter, 1980). Attention duration is one of the main measures of eye-tracking, and the longer the duration of attention to a stimulus usually indicates that the stimulus is more attractive to the consumer (Ji et al., 2023). Studies have shown that there is a general association between individual attention and visual aesthetics (Mitrovic et al., 2020), and that highly attractive faces are more effective in attracting the attention of subjects compared to less attractive faces (Valuch et al., 2015). In addition, Visual attention is often a prerequisite for the subsequent process of guiding consumers to choose a product (Behe et al., 2015). There are clear correlations between gaze and choice, and many studies have confirmed that there is a relationship between gaze allocation and choice (Armel et al., 2008; Shevlin and Krajbich, 2021). In the field of e-commerce and consumer behavior, eye-tracking techniques have been widely used, and Wang et al. (2017) found that when smile intensity is low, Duchenne smiles attract subjects’ attention to the model’s face and product information more than non-Duchenne smiles, and lead to consumers’ stronger purchase intention. Fei et al. (2021) confirmed that highly attractive streamers can immediately attract more attention, which in turn leads to more favorable responses from consumers to the information conveyed by the streamer.

Based on the above analysis, this study concludes that streamers with different physical attractiveness will cause different attention allocation situations for consumers in a live marketing situation. So the following hypothesis is proposed:

*H7*: Streamers with high physical attractiveness are able to gain more consumer attention to the streamer's face and products.

*H8*: Consumers with low involvement are more likely to be attracted by the external appearance of streamers, and their attention duration to streamers is longer.

*H9*: Consumer involvement significantly influences the relationship between streamers’ physical attractiveness and consumers’ attention duration. There is a significant difference in the attention duration that consumers with low involvement level spend on different physical attractive streamers, while there is no significant difference in the attention duration that consumers with high involvement level spend on different physical attractive streamers.

*H10*: Consumers’ attention duration is positively related to consumers response behavior.

*H10a/H10b/H10c*: Consumers’ attention duration is positively related to consumers continued watching/continued engagement/purchase intention.

## 3 Research methodology

This experiment adopts the between-group design of 2 streamers’ physical attractiveness (high/low) × 2 consumers’ involvement (high/low). This experiment simulates a real Taobao live shopping scene with the help of eye-tracking equipment. Subjects first viewed screenshots of the live broadcast and subsequently scored their continued watching intention.

### 3.1 Experimental stimulus materials

The screenshots of the live broadcast in this study were taken from Taobao Live, and in order to avoid the influence of external factors such as the name of the live room, the number of viewers, and the bullet screen, only the streamer and the product information were retained in the screenshots of this experiment. In addition, in order to reduce the potential influence of the experimental product brand on the subjects, this study used PS software to erase the brand information of the product.

First of all, lipstick was chosen as the experimental material in this study for the following reasons: first, Kahle and Homer (1985) showed that the appearance attractiveness of an endorser has a more significant effect on consumers when promoting appearance-related products (including perfume, cosmetics, and hair care products). Second, in the live-streaming marketing context, lipstick, as an experience product, can be more intuitively displayed by the streamers. Third, lipstick is a more familiar product for college students, which can reduce the impact of consumer curiosity on this experiment.

Second, in determining the streamers with high and low physical attractiveness, this study intercepted the live interfaces of four groups of streamers with high and low extrinsic attractiveness in four different e-commerce stores, and ensured that the live scene furnishings and streamers’ clothes were similar in each group of experimental materials.

Finally, a pre-experiment was conducted on the four groups to determine the final experimental material. At this stage, 30 subjects were recruited to rate the physical attractiveness of each of the four groups of streamers. The scale for measuring physical attractiveness is adapted from Crocker et al. (2003) and adjusted to delete some of the measurement items, resulting in 1 measurement questions (Table 1). The results showed that all four groups of streamer pictures were significantly different, and the most was finally selected as the stimulus material for this experiment (M _high_ = 4.45 ± 1.203, M _low_ = 1.53 ± 0.746, *t* = 10.2543, *p <* 0.001).

**Table 1:** Construct items and sources.

S. No.	Var.	Items	Statements	References
1	PA	PA1	This streamer looks attractive in appearance.	Crocker et al. (2003)
2	QI	QI1	I look forward to watching the streamer live.	Lee and Watkins (2016)
		QI2	I feel like the streamer is an old friend.	
		QI3	I hope to meet the streamer in real life.	
		QI4	This streamer makes me feel comfortable, like I am with a friend.	
		QI5	When the streamer shows me how she feels about the lipstick, it will help me decide what I think of the lipstick.	
3	FE	FE1	I had a pleasant time watching the streamer live.	Koufaris (2002)
		FE2	While watching the streamer’s live stream, I felt like time passed quickly.	
		FE3	I felt immersed while watching the streamer’s live stream.	
		FE4	I would be unaware of my surroundings while watching the streamer’s live stream.	
4	CWI	CWI1	I have a desire to continue watching the streamer live in the future.	Chen and Liao (2022)
		CWI2	I plan to continue to watch the streamer live on a regular basis.	
		CWI3	I will always try to continue to watch this streamer live.	
5	CEI	CEI1	I still come to watch this streamer’s live stream.	Algesheimer et al. (2005), Hollebeek et al. (2014), and Martins Rebouças Nery et al. (2021)
		CEI2	I would like to like and retweet the streamer’s live stream.	
		CEI3	I will be watching for the streamer’s next live time and live preview.	
6	PI	PI1	I have a high purchase intention from this streamer’s live stream.	Dodds et al. (1991)
		PI2	When I need to buy similar products, I will prioritize buying from this streamer.	
		PI3	I would recommend the products recommended by this streamer to others.	
7	CI	CI1	Lipstick is very important to me.	Zaichkowsky (1985)
		CI2	I am very interested in lipstick.	
		CI3	I will make an effort to search for information before purchasing lipsticks.	
		CI4	I would take the time to compare the differences between different brands of lipsticks.	
		CI5	I would choose the brand of lipstick very carefully.	
		CI6	I would have great fun buying lipsticks.	

PA, physical attractiveness; QI, quasi-social interaction; FE, flow experience; CWI, continued watching intention; CEI, continued engagement intention; PI, purchase intention; CI, consumer involvement; Var, variable.

### 3.2 Subjects

In this experiment, 128 college student subjects were openly recruited from a university in Hebei, including 64 males and 64 females, aged between 18 and 25 years old, with an equal proportion of males and females in each experimental group. The demographic details are given in Table 2. All subjects’ naked eye/corrected visual acuity was within the standard range, and all of them had experience in webcast shopping. Subjects were randomly assigned to four different experimental groups.

**Table 2:** Respondents profile.

Measures		Frequency	(%)
Gender	Male	64	50.0
	Female	64	50.0
Age	18–25	123	96.1
	25–35	5	3.9
Education	Undergraduate	38	29.7
	Master	90	70.3
Income	0–999	71	55.5
	1,000–1999	45	35.1
	2000–2,999	6	4.7
	3,000–above	6	4.7

### 3.3 Experimental instruments

In this study, the iView X RED telemetric eye-tracking device from SMI, Germany, was used, with a sampling frequency of 120 Hz, and the data of the subjects’ eye-tracking indexes were automatically recorded throughout the study. The experimental stimulus material was presented on a 19-inch computer monitor with a resolution of 1,280 × 1,024 pixels. Finally, the heat map of the eye-tracking experiment was plotted by Begaze 3.3, and data were examined and analyzed by SPSS 20.0 (processv3.3).

### 3.4 Experimental procedures

Before the experiment began, the subjects were first introduced to the experimental procedure, and then guided to sit about 60 cm in front of the computer screen. The subjects were also instructed to place their heads on the stand so that the subjects’ line of sight was flush with the center of the monitor, and finally the eye-tracker was calibrated and adjusted to start the formal experiment. During the formal experiment, the text description of this experimental scenario was first displayed on the screen, “You are ready to buy lipstick for a close friend, and you are ready to shop on an e-commerce live platform for the convenience of purchase at an affordable price.” Subsequently, each subject viewed the live pictures, and the eye-tracker automatically captured and recorded the subject’s eye movements at the same time. After viewing the live pictures, the subjects were invited to fill out a questionnaire, and finally, the subjects were given a reward for the experiment as a token of appreciation.

### 3.5 Questionnaire design

Before starting the experiment, subjects need to be grouped according to their level of consumer involvement. And at the end of the experiment, each subject was asked to fill out a questionnaire. Therefore, a questionnaire was designed and a seven-point Likert scale was used, with measurement dimensions ranging from complete disagreement (1) to complete agreement (7). The measurement scale of consumer involvement is adapted from Zaichkowsky (1985) and contains 6 measurement items. The measurement scale for quasi-social interaction is adapted from Lee and Watkins (2016), resulting in 5 measurement question items. The measurement scale of flow experience is adapted from Koufaris (2002), which included 4 measurement question items. Consumer response behaviors are divided into continued watching intention, continued engagement intention, and purchase intention, in which the measurement scale of continued watching intention is adapted from Chen and Liao (2022) and included 3 measurement question items; the measurement scale of continued engagement intention is adapted from the scales of Algesheimer et al. (2005), Hollebeek et al. (2014), and Martins Rebouças Nery et al. (2021), and contains 3 measurement items; the measurement scale of purchase intention is adapted from Dodds et al. (1991) and contains 3 measurement items. The adapted questionnaire is given in Table 1.

## 4 Results

### 4.1 Measurement models

Difference test were first conducted on the groupings of streamers’ physical attractiveness and consumer involvement. The results show that there is a significant difference in the physical attractiveness of streamers (M_high_ = 4.03 ± 1.699, M_low_ = 1.88 ± 0.826, *t* = 9.0129, *p <* 0.001), and the difference in consumer involvement is equally significant (M_high_ = 6.0547 ± 0.58196, M_low_ = 3.5026 ± 1.02676; *t* = 17.299, *p <* 0.001). Therefore, the experimental grouping design of this study has good operational validity.

Secondly, the reliability and validity of the scales for quasi-social interaction, flow experience, continued watching intention, continued engagement intention, purchase intention, and consumer involvement were examined, and the results of the test showed that the combined validity CR were 0.892, 0.887, 0.880, 0.897, 0.921, 0.890, the α coefficients were 0.899, 0.900, 0.928, 0.892, 0.954, 0.894, and the AVE were 0.626, 0.664, 0.805, 0.744, 0.795, 0.656. The test results showed that the scale used in this experiment had good reliability and validity.

### 4.2 Hypothesis test

#### 4.2.1 The main effect of physical attractiveness on consumer response

**Figure 2:**
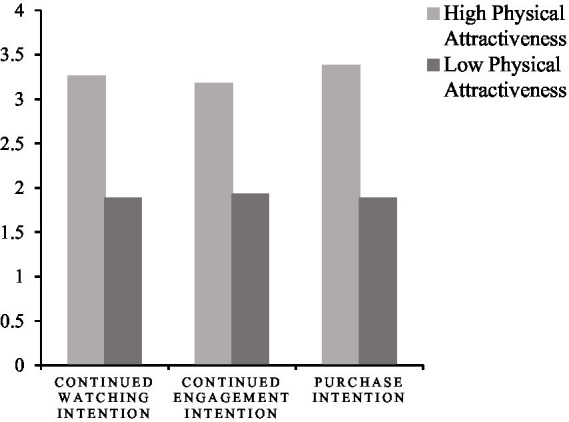
Results of the main effect on the physical attractiveness of streamers.

ANOVA was conducted with continued watching intention, continued engagement intention and purchase intention as dependent variables, respectively. The results showed that subjects in the high physical attractiveness group had active continued watching intention (M_high_ = 3.2604 ± 1.44196, M _low_ = 1.8854 ± 0.82342, *F* = 43.884, *p <* 0.001), continued engagement intention (M_high_ = 3.1771 ± 1.34383, M_low_ = 1.9271 ± 0.76858, *F* = 41.726, *p <* 0.001), and purchase intention (M_high_ = 3.3802 ± 1.65218, M_low_ = 1.8854 ± 0.68033, *F* = 44.792, *p* < 0.001). As shown in Figure 2, H1, H1a, H1b, and H1c were confirmed.

#### 4.2.2 Mediating effects of quasi-social interaction and flow experience

First, Bootstrap method was used to test the mediating effect of quasi-social interaction, and the test results were shown in Table 3. The results showed that consumer’s quasi-social interaction mediates the effect of the physical attractiveness of streamers on consumer’s continued watching intention (Indirect effect: *β* = 0.3334; LLCI = 0.1949, ULCI = 0.4614), continued engagement intention (Indirect effect: *β* = 0.2810; LLCI = 0.1746, ULCI = 0.3048) and purchase intention (Indirect effect: *β* = 0.3093; LLCI = 0.1795, ULCI = 0.4459). After adding the mediating variable quasi-social interaction, the effects of the physical attractiveness of streamers on consumers’ continued watching intention, continued engagement intention and purchase intention were still significant, which proved that quasi-social interaction partially mediates the effects between the physical attractiveness of streamers and consumers’ continued watching intention, continued engagement intention and purchase intention, which accounted for 50.6, 47.7, and 44.6% of the total effect (0.6585; 0.5895; 0.6952), respectively. H2, H2a, H2b, and H2c were confirmed.

**Table 3:** Results of the mediating effects of quasi-social interaction and flow experience.

Path	β	95% confidence interval	
		LLCI	ULCI
Physical attractiveness—quasi-social interaction—continued watching intention	0.3334	0.1949	0.4614
Physical attractiveness—quasi-social interaction—continued engagement intention	0.2810	0.1746	0.3048
Physical attractiveness—quasi-social interaction—purchase intention	0.3093	0.1795	0.4459
Physical attractiveness—flow experience—continued watching intention	0.3758	0.2515	0.5027
Physical attractiveness—flow experience—continued engagement intention	0.2796	0.1340	0.4215
Physical attractiveness—flow experience—purchase intention	0.3032	0.2467	0.5373

Second, Bootstrap method was used to test the mediating effect of flow experience, and the test results were shown in Table 3. The results showed that consumer’s quasi-social interaction mediates the effect of the physical attractiveness of streamers on consumer’s continued watching intention (Indirect effect: *β* = 0.3758; LLCI = 0.2515, ULCI = 0.5027), continued engagement intention (Indirect effect: *β* = 0.2796; LLCI = 0.1340, ULCI = 0.4215) and purchase intention (Indirect effect: *β* = 0.3032; LLCI = 0.2467, ULCI = 0.5373). After adding the mediating variable flow experience, the effects of the physical attractiveness of streamers on consumers’ continued watching intention, continued engagement intention and purchase intention were still significant, which proved that flow experience partially mediates the effects between the physical attractiveness of streamers and consumers’ continued watching intention, continued engagement intention and purchase intention, which accounted for 57.1, 47.4, and 43.6% of the total effect (0.6585; 0.5895; 0.6952), respectively. H3, H3a, H3b, and H3c were confirmed.

#### 4.2.3 Chain mediating effects of quasi-social interaction and flow experience

**Figure 3:**
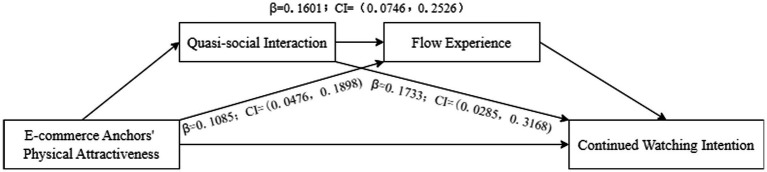
Chain mediation effect test results 1.

First, the chain mediation model of “Physical Attractiveness—Quasi-social interaction—Flow Experience—continued watching intention” was validated by using the Bootstrap method, and the results were shown in Figure 3. The results showed that the mediating effects of quasi-social interaction (*β* = 0.1733; LLCI = 0.0285, ULCI = 0.3168) and flow experience (*β* = 0.1085; LLCI = 0.0476, ULCI = 0.1898) were significant, and the hypotheses H2a and H3a were verified again. And the mediating effects of quasi-social interaction and flow experience were also significant (*β* = 0.1601; LLCI = 0.0746, ULCI = 0.2526). Therefore, H4a were confirmed.

**Figure 4:**
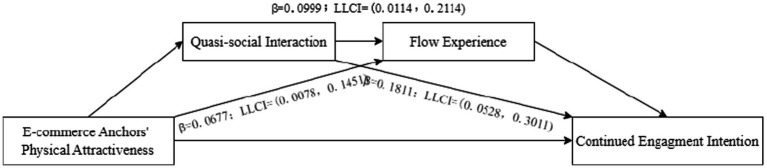
Chain mediation effect test results 2.

Second, the chain mediation model of “Physical Attractiveness—Quasi-social interaction—Flow Experience—continued engagement intention” was validated by using the Bootstrap method, and the results were shown in Figure 4. The results showed that the mediating effects of quasi-social interaction (*β* = 0.1811; LLCI = 0.0528, ULCI = 0.3011) and flow experience (*β* = 0.0677; LLCI = 0.0078, ULCI = 0.1451) were significant, and the hypotheses H2b and H3b were verified again. And the mediating effects of quasi-social interaction and flow experience were also significant (*β* = 0.0999; LLCI = 0.0114, ULCI = 0.2114). Therefore, H4b were confirmed.

**Figure 5:**
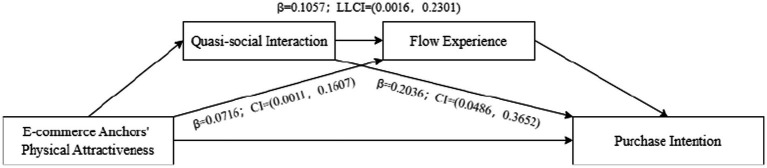
Chain mediation effect test results 3.

Third, the chain mediation model of “Physical Attractiveness—Quasi-social interaction—Flow Experience—purchase intention” was validated by using the Bootstrap method, and the results were shown in Figure 5. The results showed that the mediating effects of quasi-social interaction (*β* = 0.2036; LLCI = 0.0486, ULCI = 0.3652) and flow experience (*β* = 0.0716; LLCI = 0.0011, ULCI = 0.1607) were significant, and the hypotheses H2c and H3c were verified again. And the mediating effects of quasi-social interaction and flow experience were also significant (*β* = 0.1057; LLCI = 0.0016, ULCI = 0.2301). Therefore, H4c were confirmed.

#### 4.2.4 Moderating effects of consumer involvement

**Figure 6:**
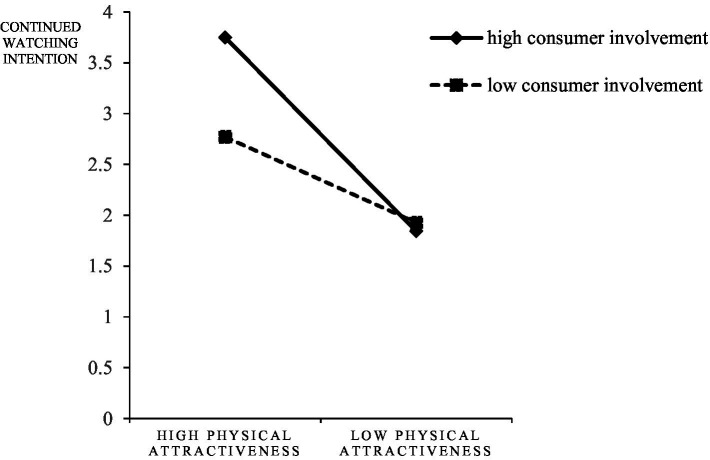
The interaction of physical attractiveness and consumer involvement on continued watching intention.

**Figure 7:**
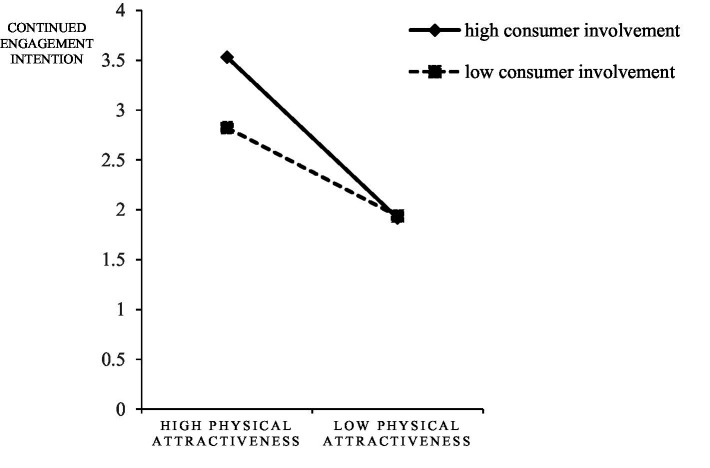
The interaction of physical attractiveness and consumer involvement on continued engagement intention.

**Figure 8:**
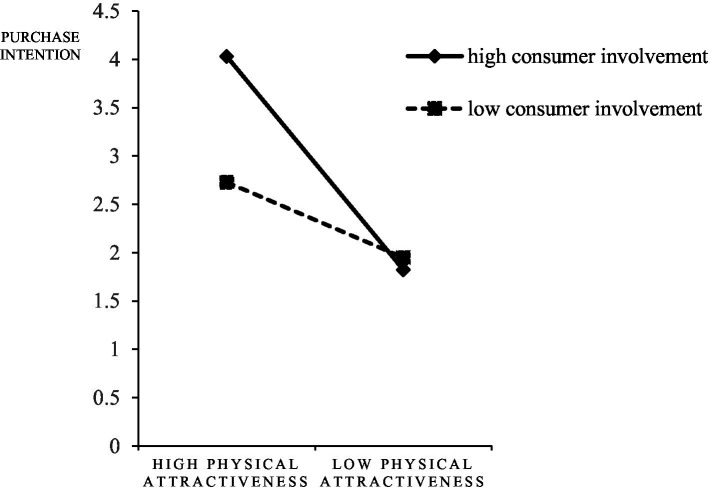
The interaction of physical attractiveness and consumer involvement on purchase intention.

First, the moderating role of consumer involvement between the physical attractiveness of streamers and consumer response was examined, and the interactions were shown in Figures 6–8. For subjects with high consumer involvement, the extrinsic attractiveness of streamers significantly affected their continued watching intention (M_HH_ = 3.75 ± 1.47135; M_HL_ = 1.8438 ± 0.91966, *F* = 38.623, *p* < 0.05), continued engagement intention (M_HH_ = 3.5313 ± 1.39856; M_HL_ = 1.9167 ± 0.82956, *F* = 31.549, *p* < 0.05), and purchase intention (M_HH_ = 4.0313 ± 1.82448; M _HL_ = 1.8229 ± 0.71334, *F* = 40.665, *p* < 0.05). For subjects with low consumer involvement, the effects of streamers’ extrinsic attractiveness on their continued watching intention (M _LH_ = 2.7708 ± 1.25134; M _LL_ = 1.9271 ± 0.72702, *F* = 10.877, *p <* 0.05), continued engagement intention (M_LH_ = 2.8229 ± 1.20627; M_LL_ = 1.9375 ± 0.71561, *F* = 12.753, *p* < 0.05), and purchase intention (M_LH_ = 2.7292 ± 1.15915; M_LL_ = 1.9479 ± 0.65094, *F* = 10.877, *p* < 0.05) were still significantly different. Therefore, H5d, H5e, and H5f were confirmed, but H5a, H5b, and H5c were not confirmed.

Secondly, the mediation analysis of flow experience under the moderation of consumer involvement was conducted through Bootstrap method, and the test results were shown in Table 4. The results showed that consumer involvement cannot effectively moderate the relationship between the physical attractiveness of streamers and consumers’ continued watching intention (Indirect effect: *β* = 0.0271; LLCI = −0.0049, ULCI = 0.0631), continued engagement intention (Indirect effect: *β* = 0.0198; LLCI = −0.0034, ULCI = 0.0490), and purchase intention (Indirect effect: *β* = 0.0201; LLCI = −0.0032, ULCI = 0.0538) through the mediation of flow experience. Therefore, H6, H6a, H6b, and H6c were not confirmed.

**Table 4:** Results of the mediating effect of flow experience moderated by consumer involvement.

Path	β	95% confidence interval	
		LLCI	ULCI
Physical attractiveness—flow experience—continued watching intention	0.0271	−0.0049	0.0631
Physical attractiveness—flow experience—continued engagement intention	0.0198	−0.0034	0.0490
Physical attractiveness—flow experience—purchase intention	0.0201	−0.0032	0.0538

#### 4.2.5 Main and interaction effects of physical attractiveness and consumer involvement on attention duration

**Figure 9:**
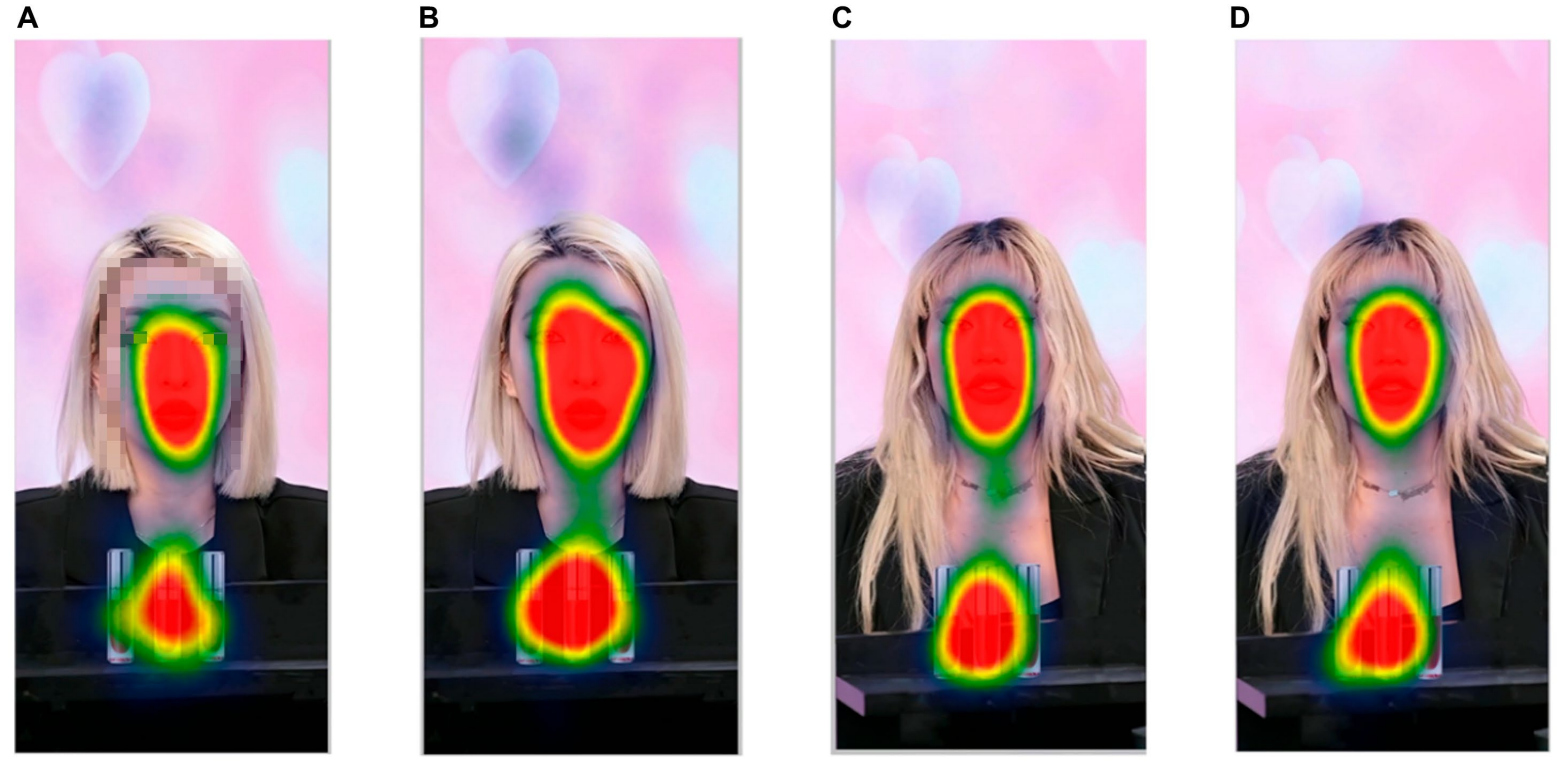
Heat map of eye-tracking experiment. **(A)** High physical attractiveness high consumer involvement. **(B)** High physical attractiveness low consumer involvement. **(C)** Low physical attractiveness high consumer involvement. **(D)** Low physical attractiveness Low consumer involvement.

**Figure 10:**
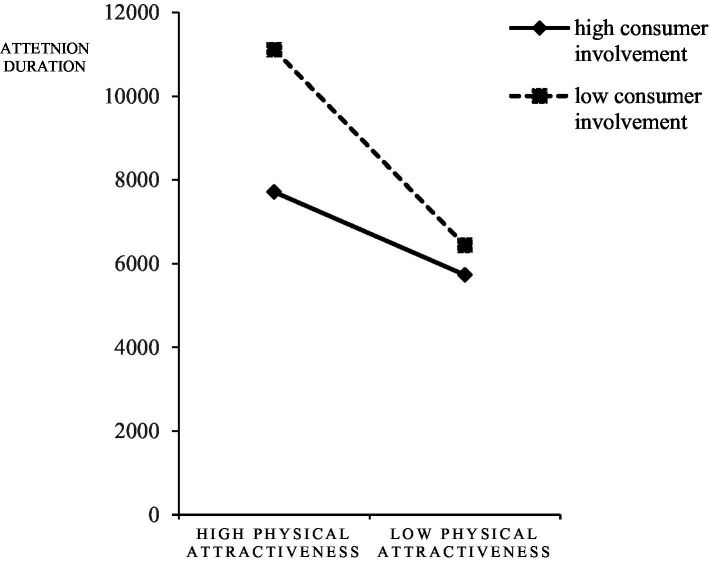
Interaction of physical attractiveness and consumer involvement on attention duration.

ANOVA of 2 physical attractiveness (high/low) × 2 consumer involvement (high/low) was conducted with physical attractiveness and consumer involvement as independent variables and attention duration as dependent variable. The test results showed that: the main effect of physical attractiveness was significant (*F* = 21.466, *p* < 0.001, $\eta_{p}^{2}$ =0.148), consumers’ attention duration for high physical attractiveness stimulus materials was longer (M _high_ = 9409.75 ± 5282.58, M_low_ = 6082.61 ± 2763.72). As shown in Figure 9, compared with the low physical attractiveness experimental group, the subjects in the high physical attractiveness experimental group were more likely to be attracted by the streamers, thus paying more visual attention; the main effect of consumer involvement was significant (*F* = 8.188, *p* < 0.05, $\eta_{p}^{2}$ =0.062), the subjects in the low consumer involvement group had longer attention duration (M_high_ = 6718.73 ± 3061.88, M_low_ = 8773.63 ± 5444.88). As shown in Figure 9, compared with the high consumer involvement experimental group, the subjects in the low consumer involvement experimental group invested more time in cognition and had a longer cognitive process; however, the interaction effect of physical attractiveness and consumer involvement was not significant (*F* = 3.505, *p* = 0.064, $\eta_{p}^{2}$ =0.027), as shown in Figure 10. Different degrees of involvement did not have a significant effect on the eye movement index, as shown in Figure 9, there is no significant difference in the allocation of attention harvested by equal physical attractiveness streamers, regardless of the degree of consumer involvement. Therefore, H7 and H8were confirmed and H9was not confirmed.

#### 4.2.6 Effects of attention duration on consumer response behavior

Regression analysis was used to test the effect of consumer attention duration on consumer response behavior, and the results showed that there was no significant effect of consumer attention duration on consumers’ willingness to watch continuously (*F* = 2.349, *p* = 0.128), willingness to participate continuously (*F* = 2.879, *p* = 0.093), and willingness to purchase (*F* = 2.273, *p* = 0.134). Therefore, H10a, H10b, and H10c were not valid.

## 5 Discussion

### 5.1 Conclusion

Based on the ELM and the Cognitive-Affective system Theory, this study introduced the characteristic variable of consumer involvement, and verified the effects of streamers’ physical attractiveness and individual involvement on consumers’ cognitive (attention duration, quasi-social interaction) and affective (flow experience) systems, as well as on consumers’ responsive behaviors (continued watching intention, continued engagement intention, and purchase intention) through eye-tracking experiments, so as to ultimately revealed the mechanisms of the streamer physical attractiveness’s influence on consumers’ decision-making. The overall results hypothesis testing in this study were show in Table 5.

**Table 5:** Hypothesis testing results.

Number	Hypothesis	Results
H1	Streamers with high physical attractiveness elicit more positive response behavior from consumers than those with low physical attractiveness.	Proved
H1a	Consumers have a more positive continued watching intention in the face of a streamer with high physical attractiveness compared to a streamer with low physical attractiveness.	Proved
H1b	Consumers have a more positive continued engagement intention in the face of a streamer with high physical attractiveness compared to a streamer with low physical attractiveness.	Proved
H1c	Consumers have a more positive purchase intention in the face of a streamer with high physical attractiveness compared to a streamer with low physical attractiveness.	Proved
H2	Quasi-social interaction mediates the relationship between streamers’ physical attractiveness and consumer response.	Proved
H2a	Quasi-social interaction mediates the relationship between streamers’ physical attractiveness and consumers’ continued watching intention.	Proved
H2b	Quasi-social interaction mediates the relationship between streamers’ physical attractiveness and consumers’ continued engagement intention.	Proved
H2c	Quasi-social interaction mediates the relationship between streamers’ physical attractiveness and consumers’ purchase intentions.	Proved
H3	Flow experience mediates the relationship between streamers’ physical attractiveness and consumers’ response.	Proved
H3a	Flow experience mediates the relationship between streamers’ physical attractiveness and consumers’ continued watching intention.	Proved
H3b	Flow experience mediates the relationship between streamers’ physical attractiveness and consumers’ continued engagement intention.	Proved
H3c	Flow experience mediates the relationship between streamers’ physical attractiveness and consumers’ purchase intention.	Proved
H4	Quasi-social interaction and flow experience act as chain mediators between streamers’ physical attractiveness and consumers’ response.	Proved
H4a	Quasi-social interaction and flow experience act as chain mediators between streamers’ physical attractiveness and consumers’ continued watching intention.	Proved
H4b	Quasi-social interaction and flow experience act as chain mediators between streamers’ physical attractiveness and consumers’ continued engagement intention.	Proved
H4c	Quasi-social interaction and flow experience act as chain mediators between streamers’ physical attractiveness and consumers’ purchase intention.	Proved
H5	Consumer involvement significantly influences the relationship between streamers’ physical attractiveness and consumers’ response behavior.	Not proved
H5a	When consumer involvement is low, Streamers with high physical attractiveness can trigger consumers more positive continued watching intention.	Not proved
H5b	When consumer involvement is low, Streamers with high physical attractiveness can trigger consumers more positive continued engagement intention.	Not proved
H5c	When consumer involvement is low, Streamers with high physical attractiveness can trigger consumers more positive purchase intention.	Not proved
H5d	When consumer involvement is high, the effect of streamers with high physical attractiveness on consumers’ continued watching intention is not significant.	Proved
H5e	When consumer involvement is high, the effect of streamers with high physical attractiveness on consumers’ continued engagement intention is not significant.	Proved
H5f	When consumer involvement is high, the effect of streamers with high physical attractiveness on consumers’ purchase intention is not significant.	Proved
H6	Consumer involvement significantly influences the relationship between streamers’ physical attractiveness and flow experience.	Not proved
H6a	Consumer involvement effectively moderates the relationship between streamers’ physical attractiveness and consumers’ continued watching intention through the mediating role of flow experience.	Not proved
H6b	Consumer involvement effectively moderates the relationship between streamers’ physical attractiveness and consumers’ continued engagement intention through the mediating role of flow experience.	Not proved
H6c	Consumer involvement effectively moderates the relationship between streamers’ physical attractiveness and consumers’ purchase intention through the mediating role of flow experience.	Not proved
H7	Streamers with high physical attractiveness are able to gain more consumer attention to the streamer’s face and products.	Proved
H8	Consumers with low involvement are more likely to be attracted by the external appearance of streamers, and their attention duration to streamers is longer.	Proved
H9	Consumer involvement significantly influences the relationship between streamers’ physical attractiveness and consumers’ attention duration. There is a significant difference in the attention duration that consumers with low involvement level spend on different physical attractive streamers, while there is no significant difference in the attention duration that consumers with high involvement level spend on different physical attractive streamers.	Not proved
H10	Consumers’ attention duration is positively related to consumers response behavior.	Not proved
H10a	Consumers’ attention duration is positively related to consumers continued watching intention.	Not proved
H10b	Consumers’ attention duration is positively related to consumers continued engagement intention.	Not proved
H10c	Consumers’ attention duration is positively related to consumers purchase intention.	Not proved

First, consumers’ attention allocation and purchase behavior decisions were influenced by the physical attractiveness of streamers, but consumers’ attention duration has no significant effect on consumers response behavior. From the perspective of consumers’ attention allocation, beautiful-looking streamers are more likely to attract consumers’ eyeballs, and can obtain more sustained attention duration, prompting consumers to stay in the live broadcasting room. The result of study on subjects’ attention duration validate selective attention theory and interpersonal attraction theory (Hamermesh, 2011; Ruffle and Shtudiner, 2015), such as the recent study by Ji et al. (2023), which found that highly attractive anchors elicit longer attention durations from consumers. In addition, this study confirmed that streamers with high external attractiveness can stimulate consumers’ more positive continued watching intention, continued engagement intention and purchase intention. This result is consistent with a recent study by Li et al. (2019), which found that service representatives with high physical attractiveness have a positive impact on consumer responses (customer satisfaction, perceived service quality, and favorability of the service representative). The same confirms the findings of Hong et al. (2019) that speakers with high physical attractiveness convey more credible and persuasive messages. This finding suggested that for a hedonic product such as lipstick, streamers with high extrinsic attractiveness are able to elicit more attention time and motivate more positive response behavior from consumers than streamers with low extrinsic attractiveness. The “beauty is good” stereotype still exists in the live marketing context, and people will still be attracted to and “pay” for beautiful things. There is no significant relationship between consumers’ attention duration and consumers response behavior, which is consistent with the findings of Ji et al. (2023), but contradicts the conclusion that there is a clear relationship between gaze and choice (Behe et al., 2015), which may be related to the fact that the present study only use attention duration as the visual data.

Second, quasi-social interaction and flow experience played a chain mediating role between streamers’ physical attractiveness and consumer responses. This study confirmed that streamers with high extrinsic attractiveness can bring more pleasant cognitive and emotional experiences to consumers, and can trigger stronger perceived prosocial interaction and flow experience, resulting in more positive consumer response behaviors. This finding suggested that streamers with high physical attractiveness can bring higher cognitive and emotional values to consumers, enabling them to immerse themselves more deeply. Good-looking streamers are more likely to generate approach behaviors that lead to a higher degree of consumer trust and preference. This finding is consistent with previous research on physical attractiveness, which suggests that physical attractiveness may have a significant impact on customers’ mood (affect), perceived value (cognition), and satisfaction, which may lead to positive behavioral intentions (Till et al., 2000; Peng et al., 2020). Similarly, it is illustrated that attractive streamers tend to generate consumer preference, admiration, love and emotional responses (Solomon et al., 2014).

Third, the moderating effects of consumer involvement on consumer response and flow experience were not significant, and the interaction effects of physical attractiveness and consumer involvement on attention duration were also not significant. This study confirmed that beautiful-looking streamers are able to elicit more positive response behaviors and longer attention duration from consumers regardless of the level of consumer involvement. After interviews with several live streaming users, it was found that for beauty products, consumers preferred the recommendations and trials of high-gloss streamers because streamers with high physical attractiveness can bring consumers a more aesthetic, pleasurable, and comfortable visual experience. The effect of “everyone loves beauty” was more significant, and thus consumer engagement failed to moderate the effect of the external attractiveness of streamers on consumers’ attention duration and response behavior. In addition, this study showed that consumer involvement failed to mediate the relationship between the physical attractiveness of streamers and consumers’ response behaviors through the mediating effect of flow experience. It was speculated that the reason for this is that consumer involvement belongs to the consumer cognitive motivational characteristic variables, which cannot clearly respond to the consumer’s personal traits, while the flow experience is influenced by the physical environment characteristics and the consumer’s characteristics (Dong et al., 2010; Cui et al., 2022; Tian and Ou, 2023), and thus the moderating effect was not significant.

### 5.2 Theoretical implications

First, this study enriches the research on physical attractiveness and reveals the mechanism by which the physical attractiveness of streamers influences consumer response behavior. Previous studies on extrinsic attractiveness have focused on service personnel (DeShields et al., 1996; Argo et al., 2008), human resources (Hamermesh, 2011), game character (Lo, 2008), and social media influencer (Masuda et al., 2022). And there is a lack of relevant studies on streamer physical attractiveness. In addition, in previous studies on streamer characteristics, scholars have mostly focused on streamer professionalism (Liu et al., 2022) and interactivity (Sun et al., 2021), and there is a lack of research related to physical attractiveness, which is most easily observed by consumers. This study takes the physical attractiveness of streamers as the research object, which expands the application boundary of the “halo effect of beauty” and the research scope of physical attractiveness.

Second, this study broadens the usage scenario of cognitive emotional system theory and further explains the changes of consumers’ internal psychological state in the context of live streaming marketing. Most of the previous studies on live streaming marketing have focused on single cognitive or emotional changes of consumers (Chen and Liao, 2022; Hsu, 2023), which still lacks a more comprehensive interpretation of consumer psychology. In contrast, this study analyzes the impact of streamers’ physical attractiveness on consumers’ response behavior in a more systematic way by exploring the intermediary mechanism of the chain of quasi-social interaction and flow experience.

Third, this study uses eye-tracking technology to further reveal the information processing and cognitive mechanisms behind the influence of streamers’ physical attractiveness on consumer behavior from the perspective of visual attention. The eye-tracking index obtained by eye-tracking technology is a relatively more objective physiological data, which can more accurately reflect the consumer cognitive decision-making process. At the same time, this study reveals the mechanism of streamers’ physical attractiveness on consumers’ response behavior through the analysis of physiological data and self-reported data, which makes the study more complete.

### 5.3 Practical implications

First of all, hire high-value streamers to attract consumers’ attention. The live broadcast room should make reasonable use of the halo effect of beauty to attract the attention of consumers, and to capture the consumer’s “love of beauty” psychology. When consumers enter the live broadcast room for the first time, what attracts their eyes most is the streamer itself, and choosing the streamer with high external attractiveness is one of the effective strategies for the live broadcast room to obtain higher sales and income. Although utilizing the beauty advantage may only bring consumers a short stay, attracting consumers is what will bring the chance of increased customer retention. Streamers should fully stimulate consumers’ quasi-social interaction and flow experience during live viewing by enhancing the attractiveness of their own image, thus triggering a positive cognitive-emotional experience for consumers. Live streaming e-commerce can also optimize the layout of the live room and improve the match between the streamer, the product and the live room in order to attract consumers by providing a comfortable visual experience.

Secondly, improve the streamer’s own quality to win the trust of consumers. Using the halo effect of beauty brings short-term benefits, and enhancing consumers’ shopping experience and satisfaction still requires the support of the streamer’s professional ability. High professionalism can provide consumers with more valuable products and related information, which in turn affects the cognitive purchasing decisions of consumers with a high degree of involvement. “Shouting sales” mode has gradually deviated from the original intention of live marketing, the lack of relevant product introduction and the pursuit of sales live with goods mode has made consumers dissatisfied; in addition, the frequent appearance of bad streamers and consumers cannot protect their rights has also made the live marketing market a mess. As a result, streamers need to improve their own professional level, focusing on product information, timely reconnaissance of market dynamics, improve product screening capabilities, improve the level of product demonstration, and then be able to win the trust of consumers with a strong professionalism.

Finally, the appropriate use of broadcasting techniques to stimulate consumer spending. Streamers can use some live broadcasting techniques to stimulate consumers’ competitive awareness, in order to briefly “shield” the influence of consumer involvement on their consumption decisions. In the live broadcast process, the streamers can use hunger marketing, purchase time limit and music play, etc., to enhance the influence of edge cues on consumer information processing, in order to stimulate consumer purchase motivation; streamers can also increase the bullet screen interaction and user lottery and other links to create a lively atmosphere of the live room, in order to enhance the degree of user participation, which in turn improves the quasi-social interaction perception and the flow experience, and use the “herd effect” to improves user retention and order conversion rate.

### 5.4 Limitations and future prospects

First, this study only explored the effect of streamers’ appearance attractiveness on consumer response behavior. Shen et al. classified attractiveness into appearance attractiveness, social attractiveness, and task attractiveness (Shen et al., 2019). Appearance is the first impression, which is the primary condition for consumers to enter and stay in the live broadcasting room, but the subsequent traffic conversion needs other charisma additions of the streamers. In future research, social attractiveness and task attractiveness can be further explored as other measurement dimensions of streamer characteristics.

Second, this study only used lipstick as the stimulus material for this experiment, which is relatively single and more targeted to the female group. Future research can verify whether the halo effect of beauty is applicable to live broadcasting scenarios of other product types by dividing different product types (hedonic products and practical products).

Third, this study only discussed the influence of the streamer on consumers as a person in the “people, goods, and field.” In the future, products (goods) and live broadcasting background (field) can be used as external stimuli to further explore their effects on consumer response behavior.

## Data availability statement

The raw data supporting the conclusions of this article will be made available by the authors, without undue reservation.

## Ethics statement

The studies involving humans were approved by the Institutional Review Board of Yanshan University. The studies were conducted in accordance with the local legislation and institutional requirements. The participants provided their written informed consent to participate in this study.

## Author contributions

XT: Conceptualization, Supervision, Writing – review & editing. ZH: Conceptualization, Data curation, Formal analysis, Methodology, Writing – original draft. XL: Conceptualization, Data curation, Methodology, Writing – review & editing.
